# Practical Notes and Formulæ

**Published:** 1881-06-20

**Authors:** 


### PRACTICAL NOTES AND FORMULÆ.

**Treatment of Chronic Cystitis.**—In a very complete article on "chronic cystitis" in the Dictionnaire Encyclopedique des Sic. Med., M. Chauvel indicates the following preparations:

| | | |
|---|---|---|
| ℞ | Turpentine | ℥ ss. |
| | Camphor | gr. xv. |
| | Ext. hyoscyamus | gr. ½. M. |

Sig. The ingredients are to be well mixed, and a piece the size of a cherry stone taken morning and evening.

Thompson frequently prescribes, with success, an infusion which he had seen an American use with benefit. It can be given in all forms of chronic cystitis:

| | | |
|---|---|---|
| ℞ | Uvæ ursi fol | ℥ j-ij. |
| | Paeeiræ. bravæ. rad | ℥ j-ij. M. |

Sig. Boil a quart and a half of water to a quart. Take from f. ℥ ij to f. ℥ iv, four or five times a day.

M. Gosselin recommends benzoic acid, to prevent the development of ammonia. He gives at first 15 grains per diem, gradually increasing to 60 and even 90 grains, without causing any trouble, save a slight parching of the throat. This treatment generally neutralizes the acidity of the urine after seven or eight days.—*Medical and Surgical Reyorter.*

**Dr. Bulkley's Ointment for Eczema.**—

| | | |
|---|---|---|
| ℞ | Ung. ricis | ℥ i. |
| | Zinci oxidi | ʒ ii. |
| | Ung. aquæ rosæ | ℥ iii. M. |

**Irritable Bladder.**—Many cases of irritable bladder, not dependent upon phosphatic deposit, may be relieved by the free use, internally, of benzoic acid. The following formula has been used many times with success in cases where direct pressure by an enlarged uterus or a general pelvic congestion alone suggested the cause:

| | | |
|---|---|---|
| ℞ | Acidie bnzoici | ʒ ii. |
| | Sodæ biborat | ʒ iij. |
| | Aq. cinnamoni | ℥ iv. |

M. Sig.—A tablespoonful every two hours till relief.—*Medical Review.*

**Quillaia Toothwash.**—

| | |
|---|---|
| Soap bark, ground | 4 oz. |
| Glycerin | 3 oz. |
| Diluted alcohol, sufficient for | 2 pints. |
| Oil of peppermint | 20 drops |

Macerate the soap-bark in the mixture of glycerin and diluted alcohol for three or four days, and filter through a little magnesia previously trituturated with the volatile oil.

**Soda Mint.**—

| | |
|---|---|
| Bicarbonate of soda | 4 drachms. |
| Aromatic spirit of ammonia | 1 ounce. |
| Spearmint or peppermint water | 16 ounces. |

Mix. Dose, from a desertspoonful to a tablespoonful for adults.

Napoleon's laxative, prescribed for him by Corvisart, was as follows:

| | | |
|---|---|---|
| ℞ | Potass. borotartrat | ℥ ss. |
| | Antimon. et potass. tart | gr. ¼. |
| | Sacchar | ℥ j. |
| | Aquæ | ℥ xv. |

M. Dose, a wineglassful frequently till it operates.—*Medical Review.*

**For Albuminuria in Pregnancy.**—

| | | |
|---|---|---|
| ℞ | Tr. ferri chloridi | 2 drachms. |
| | Potassæ chloras | 2 drachms |
| | Morphiæ murias | 1 grain. |
| | Tr. digitalis | 1 drachm. |
| | Aquæ distilat | ad. 2 ounces. |

M. Sig. A teaspoonful every six hours.—*Peoria Med. Monthly.*

**Arthritis.**—When the joints remain swolen after the subsidence of the acute symptoms, this formula has been found beneficial:

| | | |
|---|---|---|
| ℞ | Lithii bromidi | 3 drachms. |
| | Syr. zingerberis | ½ ounces. |
| | Aq. puræ | 1½ ounces. |

M. Sig. A teaspoonful three times a day.

**Eczema.**—

| | | |
|---|---|---|
| ℞ | Liq. plumbi sabacetas | 1 ounce. |
| | Glycerini | ½ ounce. |
| | Cherry laurel water | 3½ ounces. |

M. Sig. Lotion. Will be found useful in eczema characterized by great heat, redness and excessive discharges.—*Med. Gazette.*

**Syphilitic Neuralgia.**—

| | | |
|---|---|---|
| ℞ | Idoformi | 22 grains. |
| | Ex. gentianæ | |
| | Pulv. gentianæ, | aa. q.s. |
| | M. Ft. pil. No. 20. | |

Sig. Two or three to be taken daily. The above prescription is employed by Prof. Zessl in this form of neuralgia.—*Med. and Surg. Reporter.*

**Esmarch's Caustic Powder**—For removal of morbid growths is made of—

| | |
|---|---|
| Arsenious acid | 1 part. |
| Sulphate morphia | 1 part. |
| Calomel | 8 parts. |
| Pulv. gum arabic | 48 parts. |

Mix. Sprinkle thick every day upon a surface either raw or denuded of cuticle by a blister. This is said to be painless.—*Dr. E. Andrews, in Mich. Med. News.*

**The Administration of Cod-Liver Oil.**—If to each ounce of the oil are added two fluid drachms of tomato or walnut catsup, and this be well shaken when required for use, a mixture is formed which many persons have found quite palatable and to agree with the stomach better than any other form in which it had been taken. Another and not unpalatable mixture can be made and often taken readily by the patient, which consists of—

| | |
|---|---|
| Liebig's extract beef | ℥ ss. |
| Extract celery seeds | flʒ ss. |
| Vinegar | fl℥ j.. |
| Water | fl℥ ij. |
| Cod-liver oil | fl℥ v. |

Dissolve the extract of beef in water, add the vinegar and oil, shake well together with the extract of celery.—*Am. Jour. of Pharm.*

**Tully's Powder.**—

| | | |
|---|---|---|
| ℞ Cretæ prepar, | | |
| Pulv. camphoræ, | | |
| Extract glycyrrhizæ | aa ʒ xxxiij. | |
| Morphiæ sulphatis | ʒj. | M. |

Dose 5 to 10 grains.

**For the Stomach.**—The following is recommended as a good appetizer—

| | |
|---|---|
| ℞ Aqua menth pip. destill | ℥ viii. |
| Tr. gentian, | |
| Tr. aurant cort | av ℥ iii. |
| Tr. cardam comp | ℥ i. |

M. S. ℥ ss ten minutes before eating.

**Tartaric Acid in Diphtheria.**—A writer in a French journal advises tartaric acid as a local application in diphtheria. He says: The tartaric acid acting on the false membrane, changes it into a gelatinous mass and thus favors its expulsion. The formula he uses is

| | |
|---|---|
| ℞ Acid tartaric | ʒ iiiss. |
| Glycerine | ʒ ivss. |
| Aqua menth pip destill | ℥ ss ʒ iiss. |

Applications of this should be made every three hours, followed soon after by the use of lemon juice.

**Ointment of Wormwood.**—In reply to the inquiry regarding this preparation, Dr. R. Smith, Milford, Ill., kindly sends us the following note:

Ointment of wormwood, which I consider one of the best dressings extant for foul ulcers or for fresh cuts, is made as follows:

Wormwood leaves........................................ 15.0,
Lard........................................................ 35.0,
Camphor.................................................... 5.0,
Opium, in powder........................................ 1.0,
Glycerin.................................................... 15.0,
Petroleum ointment.................................... 30.0.

Add the wormwood to the lard and fry together for about one hour, and strain. Triturate together the camphor, opium, petroleum ointment; and when the lard is sufficiently cooled mix all together, adding the glycerin, and stir the ointment until cold.—*Pharmacist and Chemist.*

**Asthma.**—The following has been found very useful as a hypodermic injection in asthma—

℞ Morph. sulph.......................................... gr. iij.
Atropia sulph............................................ gr. ⅛.
Aqua laurocerasi ....................................... ℥ ss.
M. Ft. solutio.

Each syringeful of the above has one-fourth grain of morphia and one ninety-sixth atropia.

**Improved Dover's Powder.**—Dr. H. D. Vosbough, of Lyons, writes: "After trying various compounds, I have for several years used the following with results entirely satisfactory.

"In order to keep gum-camphor in a perfect powder, I grind it with an equal bulk of the English creta præparata; this I dispense as pulverized camphor. Now, my Dover's powder is compounded as follows, viz:

℞ Opii pulv.,
Ipecac pulv.................................... aa ℥ j.
Potass. nit. pulv.............................. ℥ iv.
Pulv. camph. (prepared as above noted)
Rad. glycyrrhiza pulv ........................ ℥ ij. M.

"This seems to me a better anodyne, a better sudorific, and a better hypnotic than any other compound I have ever seen called Dover's powder."—*N. Y. Med. Record.*

**Preparation for Corns.**—Jazow, in the Vratch Vedomosti, quoted by the New York Medical Record, recommends painting the corns with the following preparation: Ext. cannab. indicæ, 5.0; acid. salicyl. 20.0; collodii, 240. In all cases where it was used, the corn rapidly disappeared.

DR. J. C. BATDORF, of Mechanicsville, read an essay on the hypodermic treatment of hemorrhoids, with cases. The formula which the doctor uses is—

℞ Carbolic acid crystals.............................. ʒ ij.

Pure olive oil............................................ gtt. xxv.

Dissolve the acid by heat, and add the oil.—*Med. and Surg. Rep.*

